# A novel human mast cell activation test for peanut allergy

**DOI:** 10.1016/j.jaci.2018.03.011

**Published:** 2018-08

**Authors:** Alexandra F. Santos, Natália Couto-Francisco, Natalia Bécares, Matthew Kwok, Henry T. Bahnson, Gideon Lack

**Affiliations:** a Department of Pediatric Allergy, School of Life Course Sciences, King's College London & Guy's and St Thomas' Hospital NHS Foundation Trust, London, United Kingdom; b Peter Gorer Department of Immunobiology, School of Immunology & Microbial Sciences, King's College London, London, United Kingdom; c MRC & Asthma UK Centre in Allergic Mechanisms of Asthma, London, United Kingdom; d Immune Tolerance Network, Benaroya Research Institute, Seattle, Wash

To the Editor:

Peanut allergy (PA) has a significant effect on patients' lives, and therefore an accurate diagnosis is extremely important. Peanut-specific IgE (P-sIgE) is associated with false-positive results and overdiagnosis.^1^ Measurement of Ara h 2–specific IgE is more accurate but is associated with false-negative results. Thus a considerable proportion of patients need to undergo an oral food challenge (OFC), the current gold standard to diagnose food allergy.^2^ OFCs carry the risk of causing allergic reactions, including anaphylaxis. With the advent of new treatments for PA, use of reliable *in vitro* tests rather than OFCs to identify eligible patients and monitor clinical response to treatment is desired.

Previously, we showed that the basophil activation test (BAT) is highly discriminative between children with PA and children with peanut sensitization but not allergy (PS children) and can reduce the number of OFCs.^3^ Because the BAT requires fresh blood and 10% to 15% of individuals have uninterpretable BAT results caused by nonresponding basophils (ie, basophils that do not respond to IgE-mediated but only non–IgE-mediated stimulants),^4^, ^5^ we investigated whether the ability to elicit peanut-induced cell activation could be transferred by passive sensitization of LAD2 mast cells^6^ with patients' plasma.

Children being assessed for PA (n = 174), including 73 children with PA, 60 PS children and 41 nonsensitized nonallergic (NA) children, underwent clinical assessment, skin prick tests, blood collection for immunoglobulin measurement (by using ImmunoCAP; Thermo Fisher Scientific, Waltham, Mass), and OFCs to peanut, as previously described.^3^, ^7^ Participants were grouped as patients with PA, PS patients, or NA subjects. The allergic reaction severity was classified according to the method of Ewan and Clark,^8^ and the threshold dose was determined as the total amount of peanut protein ingested during the OFC. The study was approved by the South East London Research Ethics Committee 2. Whole blood BATs and mast cell activation tests (MATs) to peanut were performed, as previously described.^3^, ^9^

Statistical analyses were performed with SAS 9.4 software (SAS Institute, Cary, NC) and JMP Pro software, Version 13.2.1. Depending on data distribution, nonparametric Wilcoxon tests or normality-based *t* tests were used, where specified. Optimal cut points were estimated from receiver operating characteristic analyses based on logistic regression models. Relationships between mechanistic outcomes were analyzed by using stratified linear models; cubic splines were used to allow for more linear curve relationships between variables. When relationships appeared linear, Pearson correlation coefficients were reported and visualized with simple linear models and 95% CIs.

LAD2 cells expressed FcεRI and CD32 on their surfaces (see Fig E1 in this article's Online Repository at www.jacionline.org). After addition of patients' plasma, IgE was detected on the cell surface. Stimulation index (SI) IgE phycoerythrin-Cy7 was strongly correlated with plasma total IgE levels (*R*_*s*_ = 0.914, *P* < .001; see Fig E2, *A*, in this article's Online Repository at www.jacionline.org) and comparable between children with PA and PS children (*P* = .160; see Fig E2, *B*). LAD2 cells expressed lysosomal-associated membrane proteins after stimulation with peanut extract, anti-IgE, or ionomycin (see Fig E3 in this article's Online Repository at www.jacionline.org).

Plasma samples from children with PA, PS children, and NA children (see Table E1 in this article's Online Repository at www.jacionline.org) were tested in the MAT. Activation of mast cells sensitized with plasma from children with PA after stimulation with peanut extract was greater than activation of mast cells sensitized with plasma from PS children (*P* < .001) or NA children (*P* < .001; Fig 1, *A*), and the response to anti-IgE was similar (*P* = .543; Fig 1, *B*). Significant differences in mast cell activation (*P* < .001) were observed between children with PA and PS children, with similar levels of P-sIgE, for instance ranging between 0.35 and 15 KU/L (Fig 1, *C*, and see Fig E4 in this article's Online Repository at www.jacionline.org). The threshold for P-sIgE levels above which the MAT was reliable was 0.4 KU/L for P-sIgE and 0.2 KU/L for Ara h 2–specific IgE (see Fig E5 in this article's Online Repository at www.jacionline.org). The false-positive results for P-sIgE and false-negative results for Ara h 2–specific IgE are also shown in Fig E5. Patients with severe reactions had greater proportions of activated mast cells compared with patients with mild-to-moderate reactions or nonallergic patients (see Fig E6 in this article's Online Repository at www.jacionline.org). The threshold dose at which children with PA reacted during the OFC was inversely correlated with the proportion of activated mast cells (*r*_*s*_ = −0.466, *P* = .0016). We analyzed the utility of the MAT to diagnose PA and to identify allergic patients at risk of severe reactions by using receiver operating characteristic curve analyses (Table I and see Fig E7 in this article's Online Repository at www.jacionline.org).

**Fig 1:**
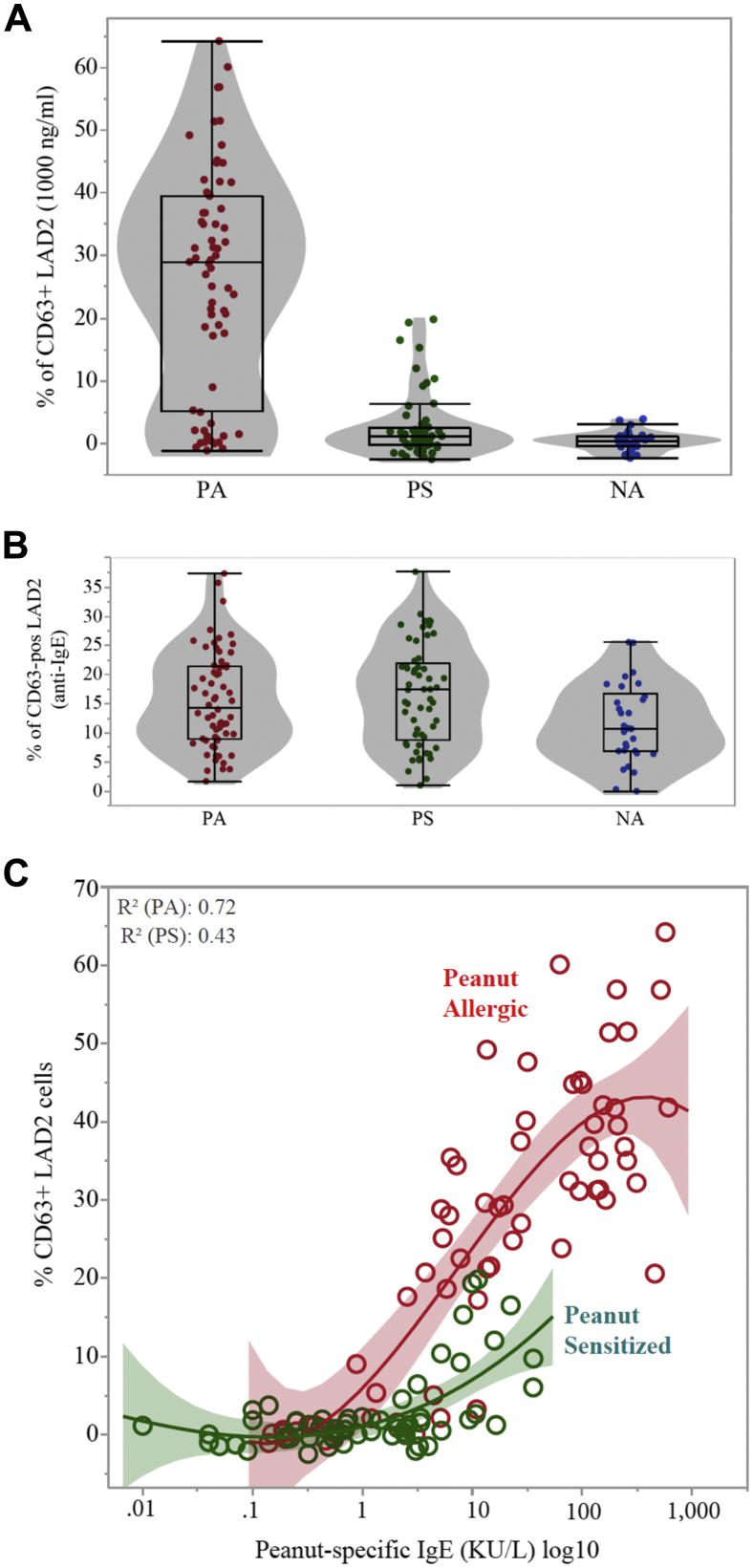
Proportion of activated LAD2 cells expressed as a percentage of CD63^+^ cells sensitized with plasma from children with PA, PS children, or NA children and stimulated with peanut extract (1000 ng/mL; **A**) or anti-IgE (1 μg/mL; **B**) and in relation to levels of P-sIgE **(C)**.

**Table I:** Diagnostic performance of the MAT

Diagnostic cutoffs Parameters	Optimal cutoff = 17.2% of CD63^+^ LAD2 cells	Cutoff to achieve 95% PPV = 17.2% CD63^+^ LAD2 cells	Cutoff to achieve 95% NPV = 0% CD63^+^ LAD2 cells	Optimal cutoff for severity = 24.8% CD63^+^ LAD2 cells
Sensitivity (%)	73 (61-82)	73 (61-82)	99 (92-100)	100 (57- 100)
Specificity (%)	98 (92-99)	98 (92-99)	18 (12-28)	87 (80-92)
PPV (%)	96 (87-99)	96 (87-99)	48 (40-56)	24 (11- 45)
NPV (%)	83 (74-89)	83 (74-89)	94 (73-99)	100 (97-100)

Ninety-five percent CIs are indicated between parentheses.

*PPV*, Positive predictive value; *NPV*, negative predictive value.

MAT results were strongly correlated with BAT results to peanut (*R*_*s*_ = 0.808, *P* < .001; see Fig E8 in this article's Online Repository at www.jacionline.org). BATs showed greater diagnostic accuracy^3^ compared with MATs, particularly because of their greater sensitivity; conversely, MATs provided a conclusive result for subjects with nonresponding basophils. Twelve children with PA had positive BAT and negative MAT results; these were patients with relatively low P-sIgE levels (median, 0.72; interquartile range, 0.27-2.79). Patients with nonresponding basophils all showed good response to anti-IgE and ionomycin and had an MAT result to peanut consistent with their allergic status.

The data reported here support the use of MATs to diagnose PA, namely in cases with equivocal P-sIgE levels, and also validate the application of the MAT as a biomarker of PA. The MAT discriminated children with PA from PS children and overcame the main limitations of the BAT because the MAT did not require fresh blood cells from the patient, thus allowing deferred testing, and provided conclusive results for all subjects with nonresponding basophils (2 of whom had PA).

Both the BAT and MAT had very high specificity when used to diagnose PA. Although the sensitivity of the BAT was superior, the enhanced specificity is the key added value of cellular tests compared with conventional serologic tests when diagnosing food allergy. The MAT can be used to diagnose PA in a sequential way when conventional tests fail, similar to what we proposed for the BAT^3^ and when it is either not possible to perform the BAT or the patient has nonresponding basophils.

Apart from its use for diagnostics, the MAT identified patients at risk of severe allergic reactions during OFCs. The sensitivity and negative predictive value of the MAT's optimal cutoff for severity was particularly high, with relatively lower specificity and positive predictive value, indicating that having a MAT result of greater than the cutoff does not necessarily mean the patient will have a severe reaction but that these patients would benefit from more intense educational measures and closer follow-up.

The MAT and the inhibition of MAT results^9^ can facilitate further study of the underlying mechanisms that determine peanut reactivity versus tolerance. This is because the MAT can be used to assess the function of allergen-specific IgE antibodies in their ability to elicit mast cell degranulation and therefore allergic symptoms, as well as the ability of antibodies of other isotypes to interfere with this effect, either by inhibiting, as shown previously for IgG_4_,^9^ or contributing to the activation of mast cells and basophils after allergen stimulation. However, this needs to be explored further. Both the BAT and the MAT are useful to test samples with equivocal P-sIgE levels to confirm PA and relay the performance of OFCs that would otherwise have positive results. Because the MAT uses plasma, which can be stored at low temperatures for long periods of time, it allows testing samples collected far from the laboratory or in the past.

The MAT is likely applicable to other food allergens. With the advent of new treatments for food allergy being approved for marketing, the MAT might prove to be a useful *in vitro* assay to monitor treatment response over time and to explore the mechanisms underlying the observed clinical changes during immunomodulatory treatments.

## Figures and Tables

**Fig E1:**
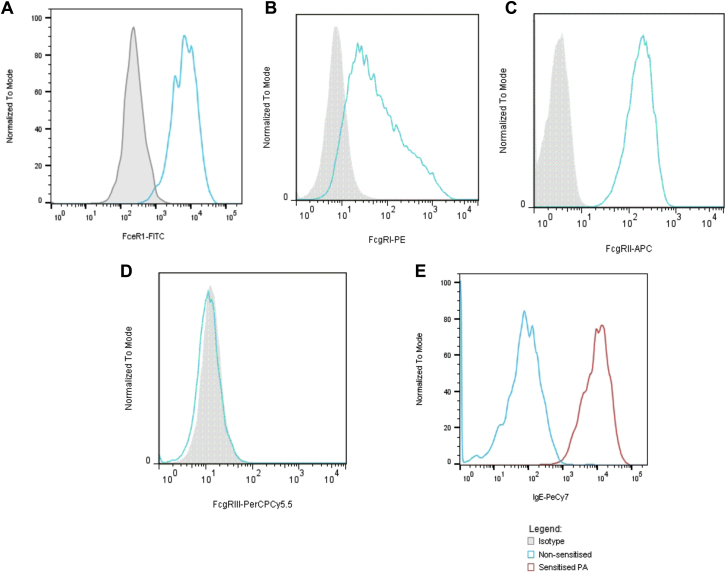
**A-D,** Expression of FcεRI (Fig E1, *A*), FcγRI (Fig E1, *B*), FcγRII (Fig E1, *C*), and FcγRIII (Fig E1, *D*) on the surfaces of LAD2 cells. **E,** IgE was detectable on the surfaces of LAD2 cells after sensitization. The *histogram in blue* represent nonsensitized LAD2 cells, and the *histogram in red* represents LAD2 cells sensitized with plasma from a patient with PA. *APC*, Allophycocyanin; *FITC*, fluorescein isothiocyanate; *PE*, phycoerythrin; *PerCP*, peridinin-chlorophyll-protein complex.

**Fig E2:**
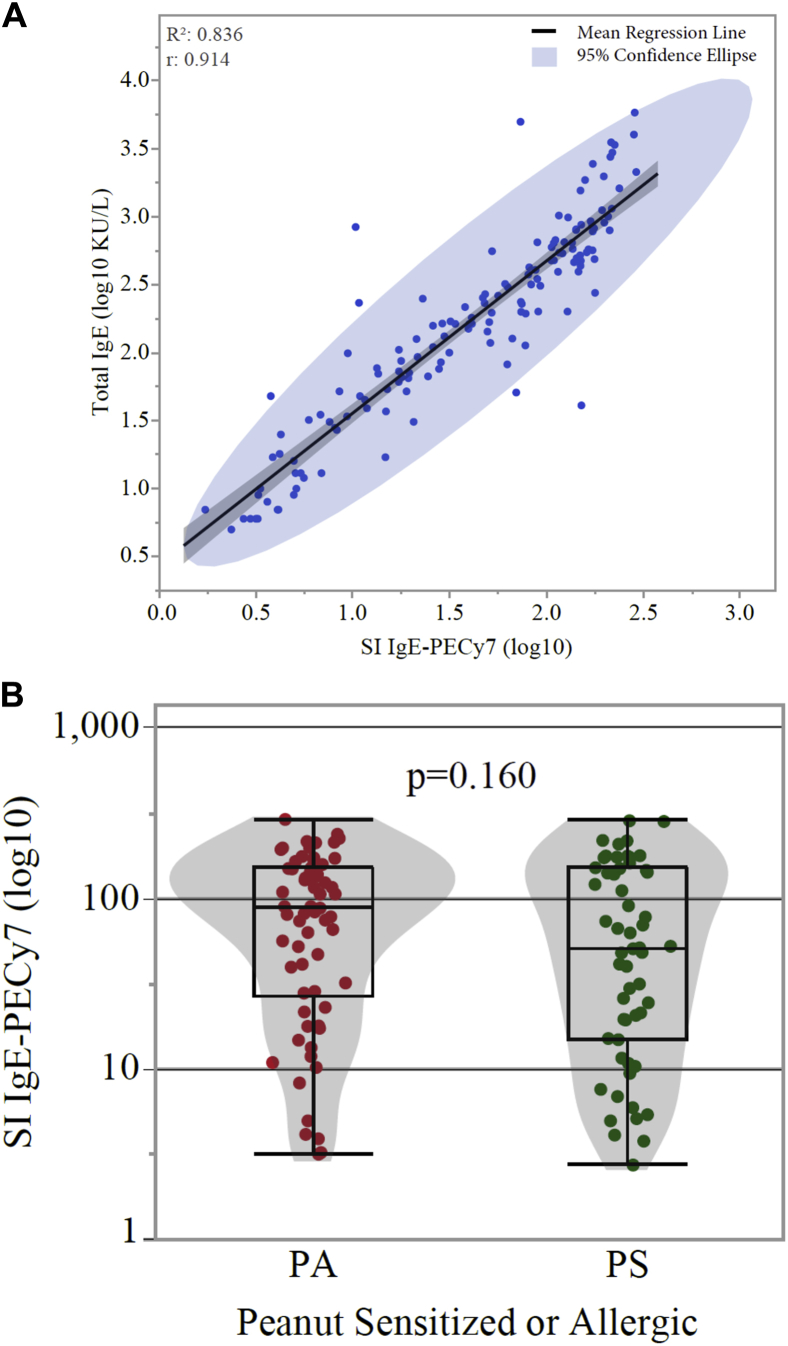
**A,** Correlation between IgE levels on the surface of LAD2 cells and total IgE levels in the plasma that the cells were sensitized with (*R*_*s*_ = 0.914, *P* < .001). **B,** Distribution of stimulation index (SI) IgE phycoerythrin-Cy7 in LAD2 cells sensitized with plasma from children with PA and PS children was not significantly different (*P* = .160).

**Fig E3:**
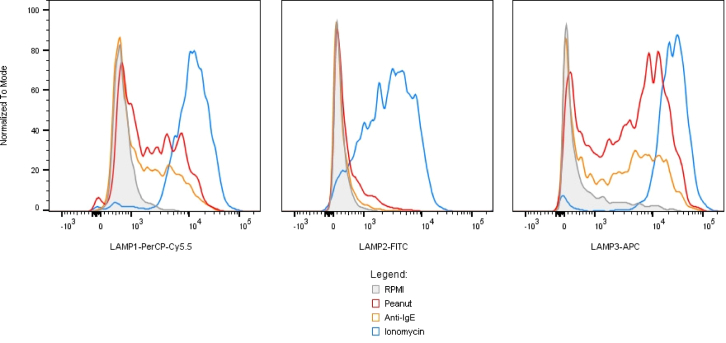
Expression of lysosomal-associated membrane proteins *(LAMPs)* on the surface of LAD2 cells after stimulation with IgE-mediated and non–IgE-mediated stimulants. LAMP-1 (CD107a) and LAMP-3 (CD63) expression increases with degranulation after stimulation with peanut extract (in red), anti-IgE (in orange), or ionomycin (in blue), whereas LAMP-2 (CD107b) expression increases with degranulation with ionomycin but not IgE-mediated stimulants. The *gray shaded area* corresponds to the negative control (ie, unstimulated cells). *APC*, Allophycocyanin; *FITC*, fluorescein isothiocyanate; *PerCP*, peridinin-chlorophyll-protein complex.

**Fig E4:**
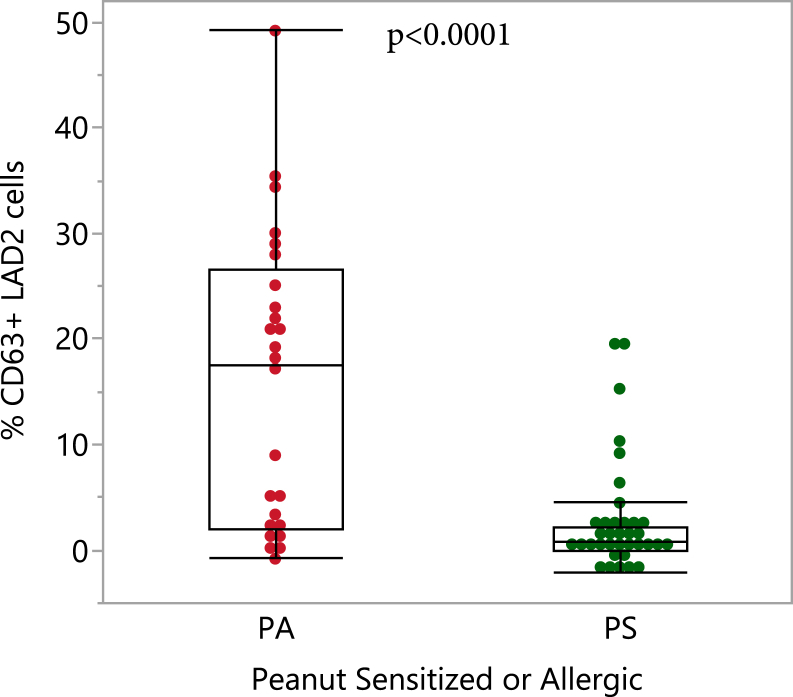
Activation of LAD2 cells sensitized with plasma samples from children with PA and PS children containing similar levels of P-sIgE ranging between 0.35 and 15.0 KU/L (which correspond to the 95% negative predictive value and 95% positive predictive value determined for P-sIgE, respectively). *PA*, Peanut allergic; *PS*, peanut sensitized tolerant.

**Fig E5:**
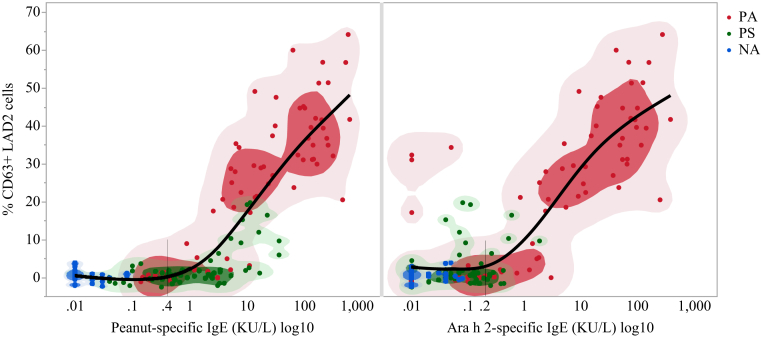
Proportion of activated mast cells sensitized with plasma samples containing varying levels of P-sIgE and Ara h 2–specific IgE. The lower limit of specific IgE above which the mast cell activation assay is reliable was estimated for peanut-specific IgE (0.4 KU/L) and for Ara h 2-specific IgE (0.2 KU/L) by observing the beginning of the inflection point from a cubic spline with a λ value of 0.8 (where the reference line intersects the smoothed regression line). *PA*, Peanut allergic; *PS*, peanut sensitized tolerant; *NA*, nonsensitized nonallergic.

**Fig E6:**
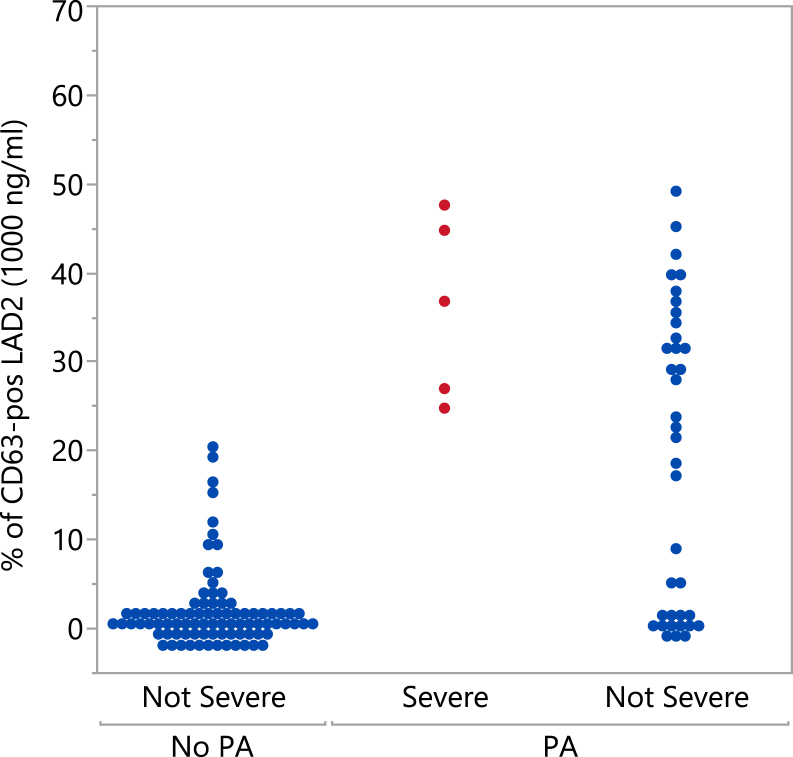
Mast cell activation to peanut extract (1000 ng/mL) in patients with no PA (in blue at *left*), patients with severe allergic reactions to peanut during the OFC (in red), and patients with PA who had mild-to-moderate reactions during the OFC (in blue at *right*).

**Fig E7:**
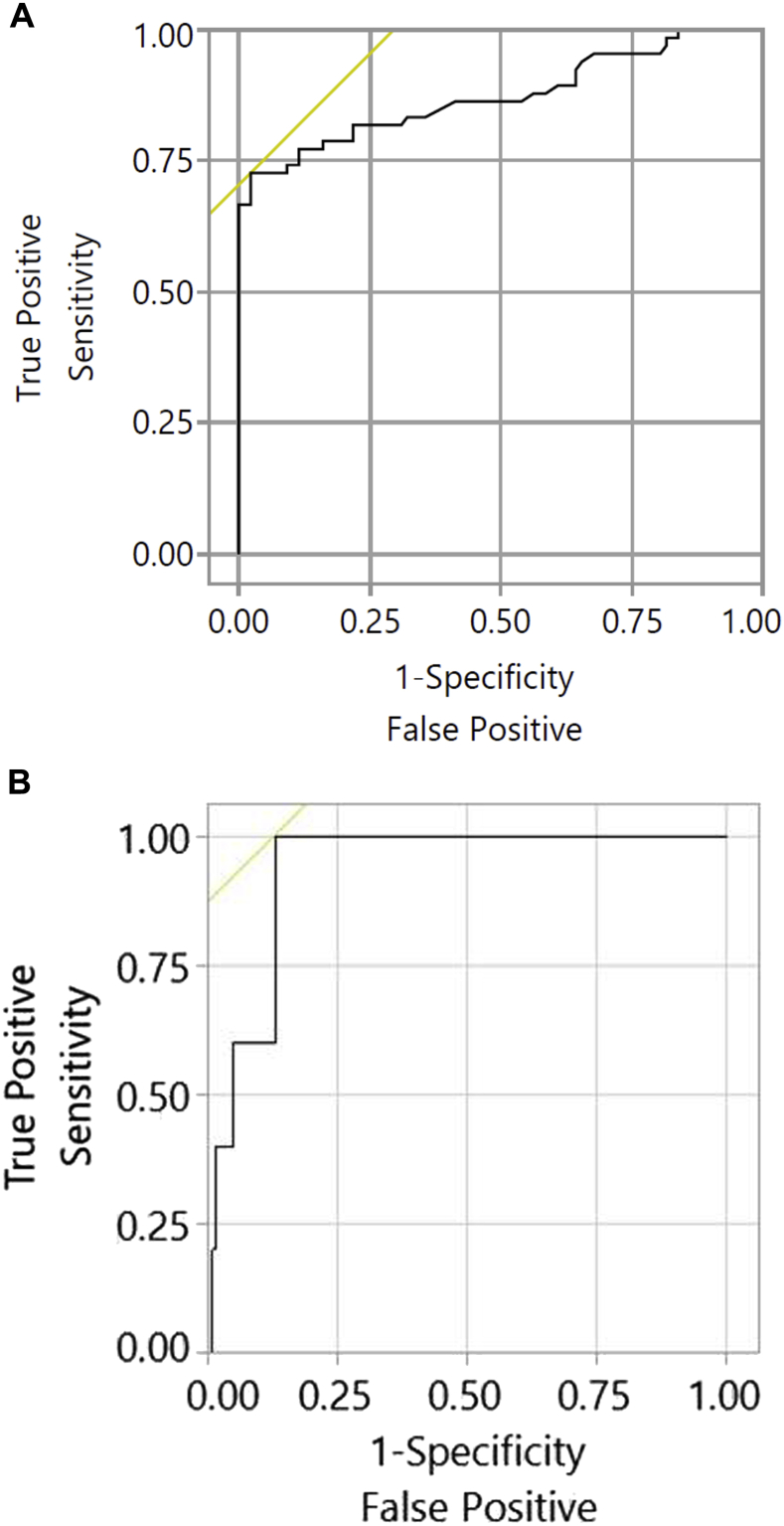
Receiver operating characteristic curve for the MAT to diagnose peanut allergy **(A)** and to identify patients at risk of severe reactions **(B)**. The overall area under the receiver operating characteristic curve was 0.874 for Fig E7, *A*, and 0.934 for Fig E7, *B*.

**Fig E8:**
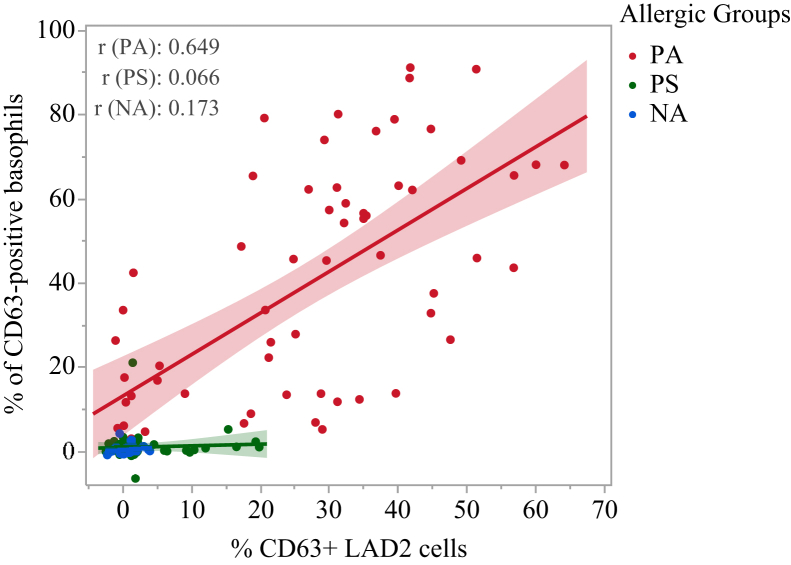
Correlation between MAT results and BAT results for the same patients. *R*_*s*_ = 0.808 and *P* < .001 for the whole population and when stratified by allergic groups: 0.649, 0.066, and 0.173 for the PA, PS, and NA groups, respectively.

**Table E1:** Antibody levels and BAT and MAT results of the study population

Median (IQR)	Patients with PA	Peanut-tolerant subjects		*P* value∗
		Peanut-sensitized but tolerant subjects	Non–peanut-sensitized, nonallergic subjects	
Total IgE (KU/L), n = 157	324 (116.25 to 759)	163 (49.5 to 572)	43.5 (12.25 to 163.75)	.0850
Specific IgE (KU_A_/L)				
Peanut, n = 173	14.05 (2.15 to 133.25)	1.10 (0.33 to 3.36)	0.01 (0.01 to 0.02)	<.0001
Ara h 1, n = 169	0.27 (0.02 to 27.4)	0.07 (0.01 to 0.34)	0.01 (0.01 to 0.01)	.0029
Ara h 2, n = 169	5.05 (0.39 to 54.5)	0.07 (0.04 to 0.17)	0.01 (0.01 to 0.03)	<.0001
Ara h 3, n = 169	0.04 (0.01 to 1.07)	0.05 (0.02 to 0.2)	0.01 (0.01 to 0.01)	.7153
Ara h 8, n = 168	0.04 (0.01 to 0.68)	0.01 (0.01 to 0.20)	0.01 (0.01 to 0.01)	.1953
Ara h 9, n = 169	0.01 (0.01 to 0.07)	0.02 (0.01 to 0.14)	0.01 (0.01 to 0.01)	.0385
BAT to peanut (% CD63^+^ basophils at 10-100 ng/mL peanut extract), n = 157	36.81 (13.06 to 62.81)	0.5 (−0.01 to 1.79)	0.2 (−0.02 to 0.68)	<.0001
MAT (%CD63^+^ LAD2 cells at 1000 ng/mL peanut extract)				
Peanut, n = 153	28.9 (5.23 to 39.55)	1.1 (−0.30 to 2.40)	0.4 (−0.43 to 1.20)	<.0001
Anti-IgE, n = 153	14.4 (8.975 to 21.53)	17.4 (8.75 to 22)	10.75 (6.8 to 16.73)	.5244

Medians and interquartile ranges are indicated. BAT and MAT results were corrected for the negative control.

*IQR*, Interquartile range.

∗ *P* values refer to the comparison between patients with PA and PS patients by using the Mann-Whitney *U* test.
